# Comparison of the cyclic fatigue resistance of ProGlider, PathGlider and One G path-finding instruments

**DOI:** 10.15171/joddd.2019.009

**Published:** 2019-04-24

**Authors:** Damla Özsu Kırıcı, Ertuğrul Karataş, Ahmet Demirhan Uygun, Ezgi Doğanay Yıldız, Kezban Meltem Çolak, Hakan Arslan

**Affiliations:** ^1^Department of Endodontics, Faculty of Dentistry, Akdeniz University, Antalya, Turkey; ^2^Department of Endodontics, Faculty of Dentistry, Atatürk University, Erzurum, Turkey; ^3^Department of Endodontics, Faculty of Dentistry, Afyon Kocatepe University, Turkey; ^4^Department of Endodontics, Faculty of Dentistry, Kırıkkale University, Kırıkkale, Turkey

**Keywords:** Cyclic fatigue, ProGlider, PathGlider, One G

## Abstract

***Background***. The aim of the present study was to compare the cyclic fatigue resistance of novel nickel titanium rotary pathfinding instruments.

***Methods***. Twenty instruments were selected for each file system. A simulated stainless steel root canal, with a 90° angle of curvature and a curvature radius of 3 mm, was used for cyclic fatigue test of the ProGlider (#16, progressive taper: 0.02‒ 0.085), PathGlider (#15, taper: .03), and One G (#14, taper: .03) instruments. Statistical analyses were performed with oneway ANOVA (P=0.05). Post hoc Tukey tests were used to determine any statistically significant differences between the groups.

***Results***. The ProGlider instruments exhibited significantly more cyclic fatigue resistance than both PathGlider and One G instruments (P<0.001). One G instruments had significantly more resistance to fracture than PathGlider instruments (P<0.05).

***Conclusion***. ProGlider instruments had better cyclic fatigue resistance than PathGlider and One G instruments.

## Introduction

The introduction of nickel-titanium (NiTi) instruments, which are commonly used in current endodontic practice, has revolutionized root canal preparation. These instruments result in less canal transportation and more dentin conservation, reduce the risk of zipping or stripping curved canals, exhibit and more elasticity and flexibility.^1-5^ Despite these advantages, NiTi instruments may fracture within the root canal without any sign of previous permanent deformation.^6-8^ Two different mechanisms have been identified for the fracture of rotary NiTi instruments: torsional failure and cyclic fatigue.^4,9^ The former occurs when the tip of the instrument binds in the canal while the shank continues to rotate, whereas the latter occurs when the instrument rotates freely in a curvature, generating tension/compression cycles in the region of maximum flexure until fracture occurs.^10,11^

Creating a glide path is an indispensable step to understanding the original anatomy of the root canal^12^ and provides less apical extrusion of debris^13^ and canal transportation.^14^ Recently, several NiTi glide-path instruments have been developed. PathGlider (Komet, Brasseler, Lemgo, Germany) is a novel single-file (#015 or #020) path-finding system with a non-cutting instrument tip and kite-shaped cross-sectional design. One G (Micro-Mega, Besançon Cedex, France) is also a single-file path-finding system, with an asymmetrical cross-sectional design. According to the manufacturer, the One G instruments have a variable pitch between each cutting edge, which limits the screwing effect.

To date, no studies have investigated the cyclic fatigue resistance of PathGlider system. Therefore, the present study aimed to compare the cyclic fatigue resistance of these path-finding instruments with that of ProGlider (Dentsply Maillefer, Ballaigues, Switzerland) and One G instruments. The null hypothesis was that there would be no difference in the cyclic fatigue resistance among the tested instruments.

## Methods

A total of 20 instruments were selected for each file system and visually inspected under a stereomicroscope at a magnification of ×20. Defective samples were discarded and new instruments without any defects replaced the defective samples.

A simulated stainless steel root canal with a 90° angle of curvature and a curvature radius of 3 mm was used for the cyclic fatigue test of the ProGlider (#16, progressive taper: 0.02–0.085), PathGlider (#15, taper: 0.03) and One G (#14, taper: 0.03) instruments. All the instruments were placed in the canal up to the center of the curvature, which was 4 mm from the tip of the file. During testing, a synthetic oil (KaVo Dental GmbH, Biberach, Germany) was sprayed into the artificial canal for lubrication. The instruments were rotated at a speed of 300 rpm and 2.5 Ncm torque with an X-Smart motor (X-Smart, Dentsply Maillefer) until fracture occurred. The time to fracture was recorded in seconds using a chronometer. The motor was operated and timing was launched after the instruments had been positioned in the artificial canals as described above. As soon as the fracture of the instrument was detected visually and audibly, timing was stopped and the time to fracture was recorded in seconds. The cyclic fatigue test of all the instruments was performed by one operator.

### Statistical Analysis

Statistical analyses were performed with one-way ANOVA (P=0.05). Post hoc Tukey tests were used to determine any statistically significant differences between the groups. All the statistical analyses were performed using SPSS 20 (IBM SPSS Inc., Chicago, IL, USA).

## Results

**Figure 1:**
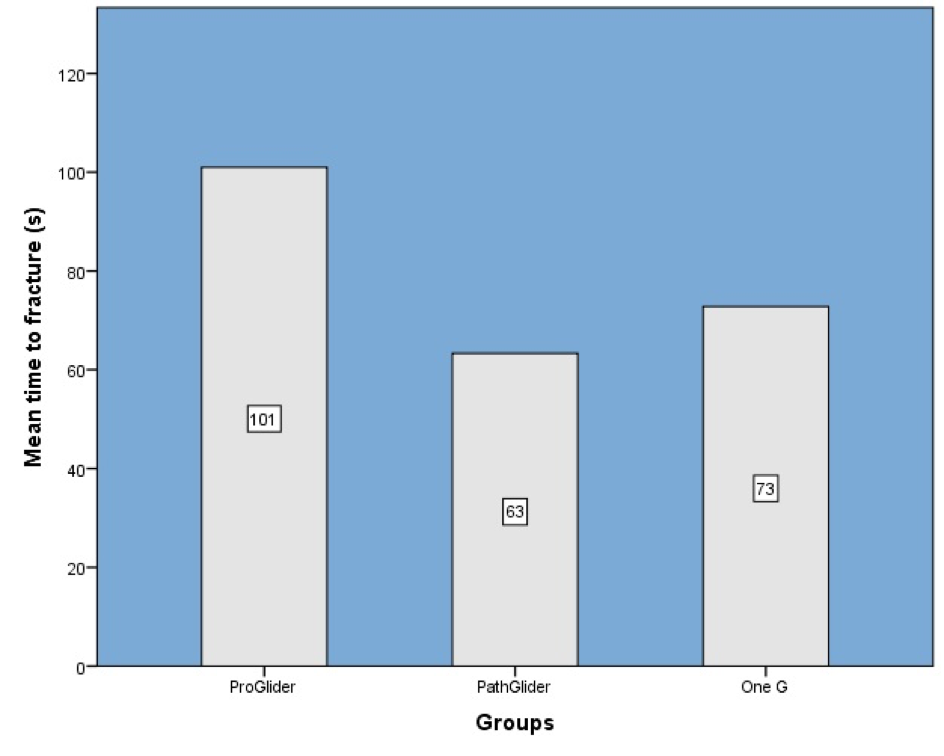
Mean times to fracture in seconds for the ProGlider, PathGlider and One G groups.

Figure 1 shows the cyclic fatigue resistance values for each group. Statistical analysis revealed significant differences between the groups in terms of mean time to fracture (P<0.05). The ProGlider instruments exhibited significantly more cyclic fatigue resistance than PathGlider and One G instruments (P<0.001). There was also a significant difference between PathGlider and One G groups. One G instruments had significantly more resistance to fracture than PathGlider instruments (P<0.05). In other words, ProGlider instruments had the highest cyclic fatigue resistance, followed by One G and PathGlider instruments.

## Discussion

The null hypothesis was refuted because there were statistically significant differences between the tested instruments in terms of cyclic fatigue resistance. According to the results of the present study, ProGlider instruments had the highest resistance to cyclic fatigue. ProGlider instruments are manufactured from a heat-treated M-wire alloy. Several studies have shown that instruments manufactured with conventional NiTi wire are less resistant to cyclic fatigue than those made of M-wire alloy.^15-19^ The difference between the manufacturing processes might explain the increased cyclic fatigue resistance of ProGlider instruments. Additionally, while ProGlider instruments have a taper of 0.02 at the first 4 mm from the tip, PathGlider and One G instruments have an 0.03 taper. In other words, the diameter of ProGlider instruments at 4 mm from the tip of the file are less than the diameter of PathGlider and One G instruments at 4 mm from the tip of the file. Previously, it has been reported that increased instrument diameter is inversely related to the cyclic fatigue life span of rotary instruments.^20^

Although PathGlider and One G files have the same taper, there was also a statistically significant difference between these instruments in terms of cyclic fatigue resistance. PathGlider instruments have a kite-shaped cross-sectional design, whereas the cross-sectional design of One G instruments is asymmetrical. Previously, it has been reported that the cyclic fatigue resistance mainly depends on the cross-sectional area and flexibility of the file.^21,22^ Differences in cross-sectional designs of these instruments might explain the present results. Moreover, the cyclic fatigue resistance of a file is also affected by its helical angle^23^ and number of threads.^24^ It can be speculated that the variable pitch design of One G instruments might have provided increased cyclic fatigue resistance. Since there are no previous studies on the cyclic fatigue resistance of PathGlider and One G instruments, a direct comparison cannot be performed.

In the current study, a standardized artificial root canal machined in a steel block was used for the cyclic fatigue test, as in several previous studies.^16,19,25,26^ The limitation of this method is that the artificial canal allows the file extreme flexibility apically at the point of the curvature, because of the width of the canal.^19^ However, the simulated artificial canal minimizes the effect of other mechanisms of instrument fracture, aside from cyclic fatigue.^27^ Since all the instruments were rotated at a speed of 300 rpm, the number of cycles to fracture were not calculated.

## Conclusion

ProGlider instruments exhibited better cyclic fatigue resistance than PathGlider and One G instruments. One G instruments had significantly more resistance to fracture than PathGlider instruments.

## Acknowledgements

The work was supported by the Atatürk University, Department of Scientific Research Projects.

## Ethical approval

This article does not contain any studies with human participants or animals.

## Conflict of interests

The authors deny any conflict of interests.

## Authors’ contributions

D.K, E.K, A.D.U, H.A designed the study. D.K, E.K., A.D.U , MC and E.D contributed to the material and method stage and writing stage. H.A and E.D analysed the data. Both authors have read and approved the final manuscript.
